# A monitor for Cellular Oxygen METabolism (COMET): monitoring tissue oxygenation at the mitochondrial level

**DOI:** 10.1007/s10877-016-9966-x

**Published:** 2016-12-20

**Authors:** Rinse Ubbink, Mark A. Wefers Bettink, Rineke Janse, Floor A. Harms, Tanja Johannes, F. Michael Münker, Egbert G. Mik

**Affiliations:** 1 Department of Anesthesiology, Laboratory for Experimental Anesthesiology, Erasmus MC – University Medical Center Rotterdam, ’s-Gravendijkwal 230, 3015 CE Rotterdam, The Netherlands; 2 Photonics Healthcare B.V., Utrecht, The Netherlands; 3 Department of Intensive Care, Erasmus MC – University Medical Center Rotterdam, Rotterdam, The Netherlands

**Keywords:** PpIX-TSLT, COMET, Mitochondrial oxygen tension (mitoPO_2_), Diagnostics, Tissue oxygenation

## Abstract

After introduction of the protoporphyrin IX-triplet state lifetime technique as a new method to measure mitochondrial oxygen tension in vivo, the development of a clinical monitor was started. This monitor is the “COMET”, an acronym for Cellular Oxygen METabolism. The COMET is a non-invasive electrically powered optical device that allows measurements on the skin. The COMET is easy to transport, due to its lightweight and compact size. After 5-aminolevulinic acid application on the human skin, a biocompatible sensor enables detection of PpIX in the mitochondria. PpIX acts as a mitochondrially located oxygen-sensitive dye. Three measurement types are available in the touchscreen-integrated user interface, ‘Single’, ‘Interval’ and ‘Dynamic measurement’. COMET is currently used in several clinical studies in our institution. In this first description of the COMET device we show an incidental finding during neurosurgery. To treat persisting intraoperative hypertension a patient was administered clonidine, but due to rapid administration an initial phase of peripheral vasoconstriction occurred. Microvascular flow and velocity parameters measured with laser-doppler (O2C, LEA Medizintechnik) decreased by 44 and 16% respectively, but not the venous-capillary oxygen saturation. However, mitochondrial oxygen tension in the skin detected by COMET decreased from a steady state of 48 to 16 mmHg along with the decrease in flow and velocity. We conclude that COMET is ready for clinical application and we see the future for this bedside monitor on the intensive care, operating theater, and testing of mitochondrial effect of pharmaceuticals.

## Introduction

Because of the importance of adequate tissue oxygen supply, many techniques have been developed for measuring oxygen in vivo over the last decades [1, 2]. The ultimate goal of measuring oxygen at the level of the mitochondria has recently become reality. We introduced the protoporphyrin IX-triplet state lifetime technique (PpIX-TSLT) for measuring mitochondrial oxygen tension (mitoPO_2_) in 2006 [3]. In the mean time, the technique has been proven to be useful in isolated cells, isolated organs and in vivo in animal studies [4–7].

PpIX-TSLT is based on the principle of oxygen-dependent quenching of the excited triplet state of protoporphyrin IX (PpIX). Application of the porphyrin precursor 5-aminolevulinic acid (ALA) induces PpIX in the mitochondria where it acts as a mitochondrial located oxygen-sensitive dye. After photo-excitation with a pulse of green light PpIX emits delayed fluorescence of which the lifetime is inversely related to the amount of oxygen. The technique is non-invasive and can be safely used in humans [8].

The ability to measure optically intracellular oxygen is providing the possibility to assess oxygenation at the end of the oxygen cascade [3, 9]. Measurements in the intracellular compartment are complementary to for example hemoglobin-based oxygen measurements. Pulse-oximetry typically measures at the arteriolar side of the microcirculation [10] while near-infrared and visible light spectroscopy are biased toward the venous compartment [11, 12]. Interstitial oxygen measurements with e.g. oxygen electrodes measure close to the cellular compartment, but are cumbersome and tissue destructive [1]. Measuring at the end of the oxygen cascade is important since (pathologic) shunting in the microcirculation or the development of tissue edema can cause cellular hypoxia, which is otherwise not detectable [13].

Besides measuring mitoPO_2_ PpIX-TSLT also provides the possibility to get insight in local tissue oxygen consumption at the mitochondrial level [14]. Mitochondrial oxygen consumption (mitoVO_2_) can be estimated by measuring the oxygen disappearance rate (ODR) in the measuring volume [15]. Recently we have demonstrated that this enables bedside non-invasive monitoring of an important aspect of mitochondrial function in animal models of critical illness [16, 17].

A clinical device featuring PpIX-TSLT has now been developed and recently entered use in clinical trials in our institution. This monitor is called “COMET”, an acronym for Cellular Oxygen METabolism. The COMET measuring system enables physicians to measure oxygen tension and oxygen consumption at the subcellular level in the mitochondria. This paper is the first description of this CE-marked device (Photonics Healthcare, Utrecht, The Netherlands). It provides the technical background, the construction of the device, and its use together with two examples of measurements in human skin.

## Methods

### Background of PpIX-TSLT

Protoporphyrin IX (PpIX) is the final precursor of heme in the heme biosynthetic pathway and is synthesized inside the mitochondria [13]. The conversion of PpIX to heme in the mitochondria is a rate-limiting step. Therefore, administration of the porphyrin precursor 5-aminolevulinic acid (ALA) enhances the mitochondrial PpIX concentration [18]. Administration of ALA does not only enhance PpIX to detectable levels, but it also ensures mitochondrial origin of the delayed fluorescence signal [3, 6, 7].

Delayed fluorescence can be observed after pulsed excitation of PpIX as delayed luminescence with the same spectrum as prompt fluorescence (red light). In contrast to prompt fluorescence delayed fluorescence has a lifetime of tens to hundreds of microseconds [3]. Delayed fluorescence is the result of photon emission due to spontaneous relaxation of the excited triplet state via bi-directional intersystem crossing. Oxygen is a very effective quencher of the excited triplet state. In the process of quenching, energy is transferred to oxygen and PpIX relaxes to the ground state without emission of a photon. This causes the lifetime of the triplet state, and thus the lifetime of the emitted delayed fluorescence, to be oxygen-dependent.

The delayed fluorescence lifetime is inversely proportional to the amount of oxygen according to the Stern–Volmer Equation [8, 19]. With the assumption of a homogenous distribution of oxygen this relationship can be used to calculate the mitochondrial oxygen tension:

1 $$MitoP{{O}_{2}}=\frac{\frac{1}{\tau }-\frac{1}{{{\tau }_{0}}}}{{{k}_{q}}}$$

where τ is the measured delayed fluorescence lifetime, τ_0_ is the delayed fluorescence lifetime in the absence of oxygen (i.e. the lifetime of spontaneous relaxation), and k_q_ is the quenching constant.

### Signal analysis

Oxygen however is heterogeneously distributed in tissues in vivo. Previous studies have shown that this also applies for mitoPO_2_ [6, 7, 13]. Delayed fluorescence from a heterogeneous system does not decay mono-exponentially, but the signal contains a lifetime distribution. Fitting Eq. 1 to a distribution of lifetimes generally leads to an underestimation of the mean PO_2_ in the measuring volume [20]. A much better estimation of the mean PO_2_ can be found by alternatively fitting a distribution of quencher concentration to the delayed fluorescence signal. The fitting function for a simple rectangular distribution with a mean mitoPO_2_ Q_m_ and a mitoPO_2_ range from Q_m_−δ till Q_m_+δ is [21]:

2 $${{Y}_{R}}(t)=\exp \left( -\left( \frac{1}{{{\tau }_{0}}}+{{k}_{q}}\left\langle mitoP{{O}_{2}} \right\rangle \right)t \right)\frac{\sinh ({{k}_{q}}\delta t)}{{{k}_{q}}\delta t}$$

where Y_R_(t) is the normalized delayed fluorescence data, <mitoPO_2_> is the mean mitoPO_2_ within the sample volume and t is the factor time.

Fitting of Eq. 2 is fast and very robust when applied to weak delayed fluorescence signals and noisy real world signals. In a previous analysis we have shown that fitting Eq. 2 allows for reliable retrieval of mitoPO_2_ values from data with signal-to-noise ratios (SNR) as low as 10 [5]. For time-domain delayed luminescence measurements SNR is defined as the maximum amplitude of the delayed fluorescence divided by the maximum amplitude of the noise. Generally a SNR above 20 is well achievable and the noise-induced error in the measurement remains below 2%.

For analysis of the delayed fluorescence signals COMET uses Eq. 2 to calculate mean mitoPO_2_ in the measuring volume under the probe. The absolute value for mitoPO_2_ is directly displayed on the screen without further processing. COMET also evaluates the signal quality, which is calculated from the SNR value; an increase of one in SNR is approximately 1% in signal quality up till a SNR of 50. Beyond a SNR 50 the increase in signal quality percentage will flatten out. As long as SNR is within an acceptable range, a SNR greater than five, COMET will show a percentage and a calculated mitoPO_2_. If COMET cannot detect a signal, or SNR is too low, less than or equal to five, the used version of the firmware makes COMET to display “no signal found” and to provide the unrealistic value of “999”.

### Monitor description

**Fig. 1:**
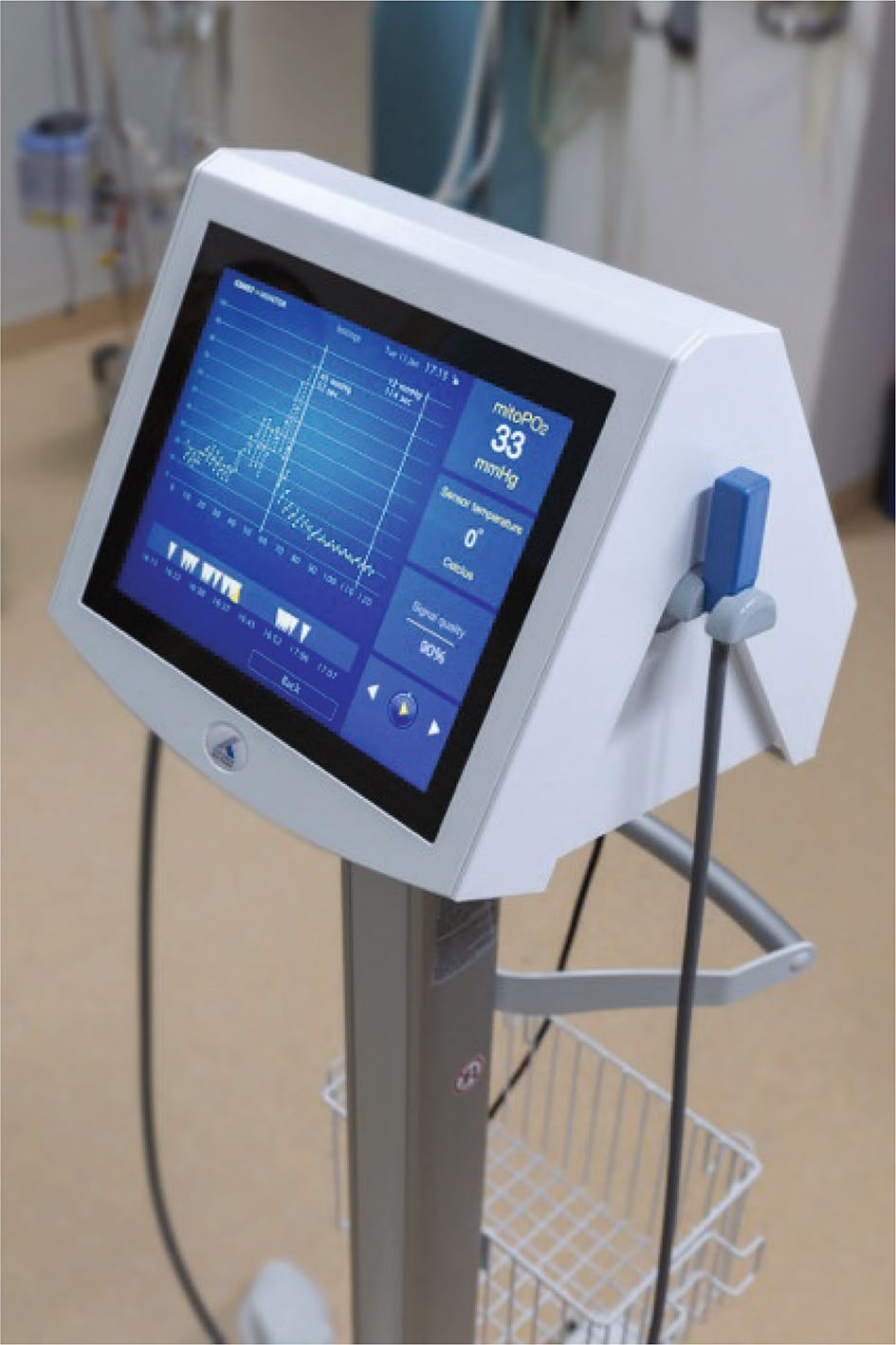
COMET monitor and skin sensor

The COMET is a medical device and class IIa classified according to the Medical Device Directive 93/42/EEC. The legal manufacturer is Photonics Healthcare B.V., Utrecht, The Netherlands. It weighs 10 kg and sizes 22 × 33 × 29 cm without cradle and port cover on the side. The COMET measurement system exists of two components shown in Fig. 1. The first component is the monitor which includes the multi-touch screen integrated user interface, light source, detection system and processing units. The second component is the COMET Skin Sensor developed for use on the human skin.

#### Hardware

The COMET is an electrically powered system (rated power consumption of 250 W). The light source and the detection system are the two core components. A 515 nm pulsed laser, pulse duration 60 ns, with a 10 Hz repetition rate illuminates the intra cellular accumulated PpIX. The fluorescent signal is projected on a gated red-sensitive photomultiplier tube. Users can interact via a multi-touch 12″ TFT-LCD screen. Apart from the main switch to turn on the device, the COMET has no physical buttons. If a USB storage device is inserted in the USB-port on the rear panel the data is exported in a comma separated file format for further processing in programs like MS Excel. The COMET can be used on a flat surface or be mounted on a trolley or arm through a VESA 75/100 compatible adapter plate.

#### Software

Lifetimes of the raw data are calculated on an embedded control board. The embedded calculation software is written in C code to simplify the development process as per IEC 62304 as required for certification. The user interface (UI) is running on a separate Linux based operating system to enhance device usability experience.

There are three different types of measurement to distinguish, shown in Table 1.

**Table 1:** The COMET different measurement types

Single measurement	One measurement per activation of the touchscreen non physical button
Interval measurement	The COMET will measure in a set interval: at the start of the interval a measurement is done, and the interval time can be chosen (60, 20, 5, 1 min)
Dynamic measurement	The COMET can conduct a series of up to 120 measurements, one measurement per second

### Location of the measurement

The COMET measures oxygen tension in mitochondria by measuring the triplet-state lifetime of PpIX. Under normal (non-sensitized) conditions PpIX is present in very low concentrations in the human skin and not detectable with the COMET. This can be overcome by the exogenous administration of ALA that leads to higher concentrations of PpIX in the mitochondria.

ALA synthase is the first and the rate-limiting enzyme of the porphyrin synthetic pathway. Under normal conditions the level of heme synthesis and the intracellular concentration of PpIX are mainly regulated by heme control of the ALA synthase activity. As a small molecule, ALA penetrates the stratum corneum [22]. Exogenously provided ALA bypasses the negative feedback controls in the heme biosynthetic pathway and leads to overproduction of PpIX [23].

The COMET can measure in healthy skin as well as in skin lesions. After ALA application the measurement area needs to be covered, to avoid consumption of PpIX by light. A priming time for ALA, typically 4 h or more, is needed to synthesize a suitable concentration of PpIX to enable measurements of mitoPO_2_ and oxygen disappearance rate.

After topical administration on healthy skin, PpIX is synthesized in the epidermis but not (significantly) in the dermis [24]. While ALA penetrates into the dermis the heme-synthesis pathway in most dermis cells is inactive. The conversion of ALA to PpIX requires energy and an intact heme cycle, thus PpIX is not synthesized in the metabolically inactive cells of the stratum corneum. In healthy skin this limits the intradermal measurement location and signal origin of the COMET to the epidermis with a thickness of about 0.1 mm [25].

**Fig. 2:**
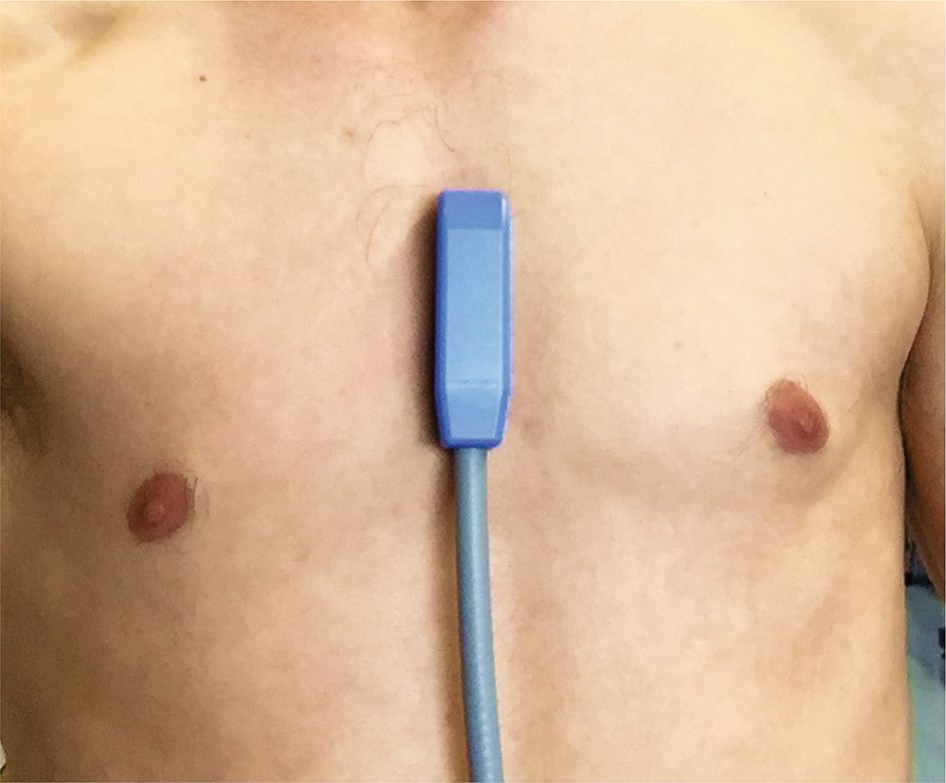
COMET skin sensor position on the sternum

The recommended measurement location is the skin of the sternum seen in Fig. 2. This provides a central measurement location less influenced by temperature changes, movement and peripheral vasoconstriction [26].

### Skin sensor

**Fig. 3:**
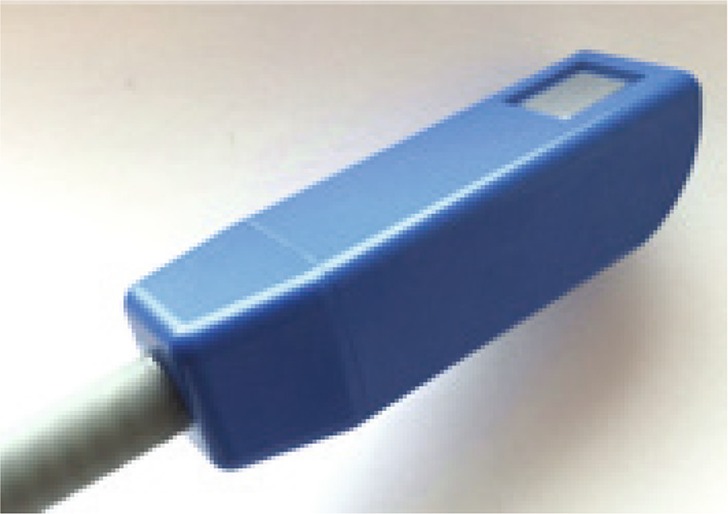
Detailed view of skin sensor

The biocompatible housing (70 × 20 × 20 mm) of the Skin Sensor, shown in Fig. 3, holds two optical fibers; the excitation and the detection fiber. A flexible metal tube protects the vulnerable optical fibers against external mechanical forces. The optical design of the sensor can collect light at approximately a right angle to the sensor cable. The light emitted by the sensor is divergent and safe for eyesight at any distance.

Ambient light entering the detection path might overload or even damage the photomultiplier tube. For protection, a photodiode in the Skin Sensor determines the ambient light before each measurement. Sensor temperature, used as an approximation of skin temperature, is measured with an electrical resistive sensor.

### Oxygen-consumption measurement

The COMET provides the opportunity for measurements in dynamic situations by taking a series of 120 samples of the mitoPO_2_ acquired at 1 Hz. This can be used to determine the oxygen disappearance rate (ODR) and reperfusion. Typically, mitochondrial oxygen availability is measured for 10–20 s in an undisturbed and stable situation. Subsequently light pressure is applied with a hand onto the sensor, to give occlusion of the microcirculation and stop local blood flow in the measurement volume, for about 45 s. After these 45 s the pressure is released and restoration of microcirculatory blood flow and mitochondrial re-oxygenation will appear.

Previously, we have described the fundamental principles behind the technology and have provided a working implementation of the technique for mitoVO_2_ measurements in vivo [14]. In summary, the ODR is generally dependent on two factors; oxygen consumption (VO_2_(t)) and Diffusive Oxygen Influx into the measurement volume (DOI(t)). The method to calculate ODR from the mitoPO_2_ kinetics is:

$$ODR=dP{{O}_{2}}/dt=-V{{O}_{2}}(t)+DOI(t)$$

The VO_2_(t) is oxygen-dependent and, according to Michaelis–Menten kinetics, can be described as:

$$V{{O}_{2}}=({{V}_{max}}{\cdot}P{{O}_{2}}(t))/({{P}_{50}}+~P{{O}_{2}}(t))$$

where V_max_ is the not supply-dependent maximal tissue oxygen consumption and P_50_ is the PO_2_ at which cellular oxygen consumption is reduced to 1/2 V_max_. PO_2_(t) denotes the PO_2_ in the measurement volume at time point t.

### In human measurements

Using COMET a dynamic measurement was performed on a healthy volunteer. Preceding the measurements an ALA plaster 4 cm^2^ (2 mg 5-amino-4oxopentacid/cm^2^) was applied for approximately 10 h (overnight) onto the skin of the sternum. A baseline of 20 s was measured before the microcirculation was occluded. The microcirculation occlusion was accomplished by application of external pressure by hand on the Skin Sensor.

Secondly, we report an incidental finding we made during an ongoing feasibility study of the COMET. The study is performed in accordance with the declaration of Helsinki and patients are consented with a protocol approved by local ethics committee METC (CCMO number NL51937.078.15). This study is set up to determine the applicability, stability, and reproducibility of the COMET measurement over a longer period of time during neurosurgery. The shown incidental finding is an observation that occurred during non-protocolled administration of the central alpha-receptor agonist clonidine.

MitoPO_2_ was measured intraoperatively, simultaneously to tissue oxygenation saturation and perfusion parameters (O2C, oxygen to see version 2424, Lea Medizintechnik GmbH, Germany). The O2C measures three parameters: The local capillary venous saturation (SO_2_), the local velocity of blood given in velocity units (VU) and the local micro vascular blood flow given in flow units (FU). Both the COMET Skin Sensor and the O2C probe (LFX-43) were positioned on the sternum next to each other.

## Results

### Oxygen-consumption measurement

**Fig. 4:**
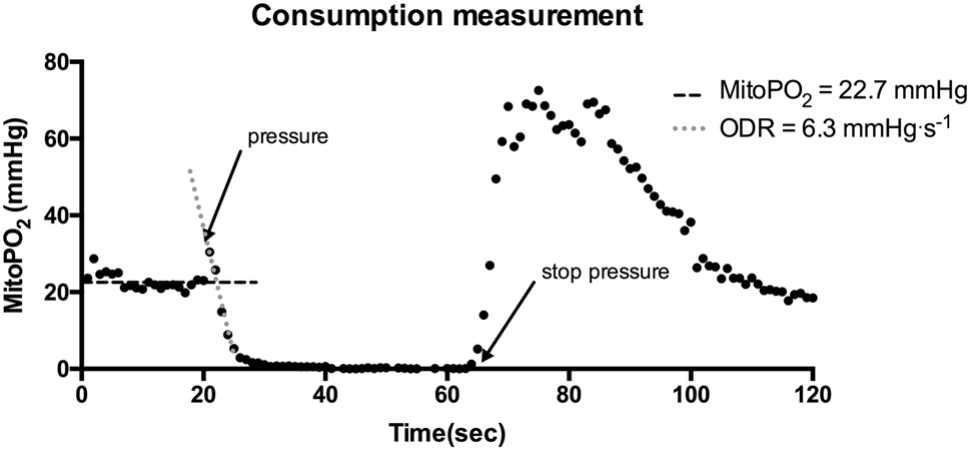
A typical dynamic measurement of mitochondrial partial oxygen pressure (mitoPO_2_) by COMET measurement system. A sample of 120 s is shown. In the first 20 s the baseline was determined, afterwards light pressure was applied on the sensor to stop microcirculation and the oxygen disappearance rate (ODR) was measured. At 60 s pressure was released

A typical example of an oxygen-consumption measurement on the healthy volunteer is shown in Fig. 4. Mean mitoPO_2_ (t_0−19_) gave a baseline mitoPO_2_ of 22.7 ± 2.1 mmHg (mean ± SD). After 20 s direct pressure with the probe was given to occlude microvascular blood flow in the skin. The available oxygen was consumed and resulted in an oxygen disappearance rate of 6.3 mmHg s^−1^. When the pressure was released and direct oxygen recovery up to 60–70 mmHg in mitoPO_2_ was seen. At 120 s the mitoPO_2_ returned to baseline values.

### Incidental finding during ongoing clinical study

**Fig. 5:**
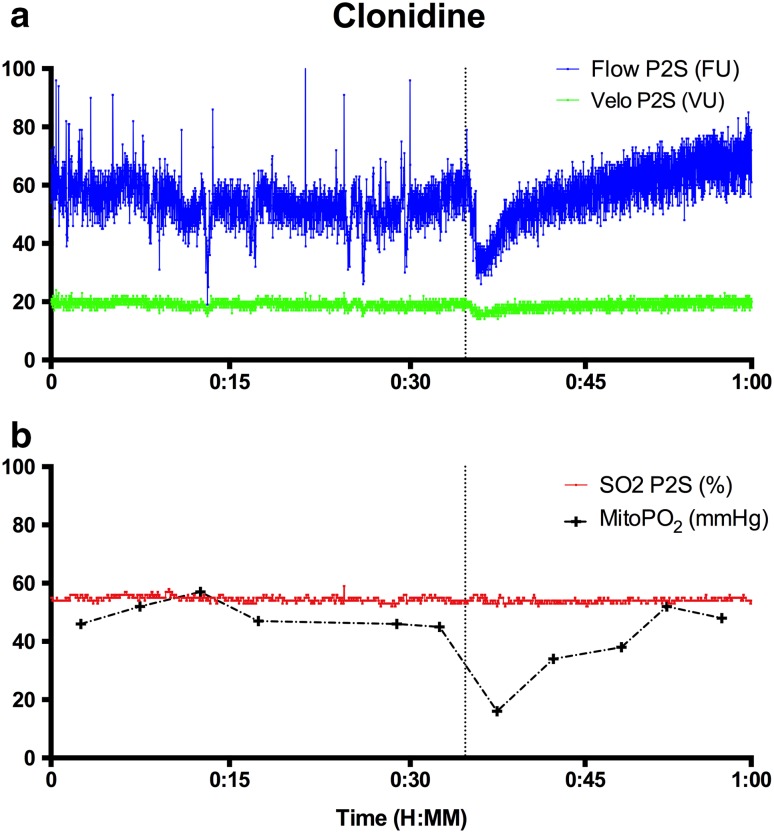
**a** Flow in arbitrary flow units (FU) and arbitrary velocity in velocity units (VU) of microcirculation as measured with probe 2 (P2S) O2C. **b** Capillary venous saturation (SO_2_)** as measured by O2C and mitochondrial partial oxygen pressure (mitoPO_2_) as measured by the COMET

In one of the measured patients during an ongoing feasibility study in neurosurgery patients, clonidine was given intravenously due to persistent hypertension. Clonidine is a central inhibitor of noradrenergic neurotransmitter transmission but also a peripheral α1-agonist. Given in a short period of time clonidine leads to initial peripheral vasoconstriction, followed by a slow onset of vasodilatation. In this particular case 150 µg of clonidine was given as a bolus application. A direct onset effect of vasoconstriction on flow and velocity but not on capillary venous oxygen saturation (SO_2_) was seen as measured by O2C Fig. 5. Flow decreased by 44% and velocity by 16%. Although SO_2_ did not change, a transient drop in mitoPO_2_ was measured with the COMET. MitoPO_2_ dropped from a steady state of 48 to 16 mmHg. After the fast clonidine administration the restoration of blood flow and velocity, mitoPO_2_ returned to baseline in approximately 15 min.

## Discussion

The COMET measurement system is the successor of the first clinical prototype previously described in this journal [17]. This new clinical monitor is safe to be used, easy to transport and applicable for in vivo measurement of mitochondrial oxygen tension and consumption in the human skin. The sternal skin is an easy accessible and non-invasive measurement location. The clinical significance of measuring in the skin arises from the fact that, like the gut, the skin can be regarded as a canary of the body [27]. The idea is that cutaneous mitochondrial PO_2_ changes foretell changes in other vital organs and systemic parameters. Indeed, in an animal model mitoPO_2_ appeared an earlier indicator of approaching the limit of physiological compensation during hemodilution than e.g. venous saturation and lactate [27, 28]. The “canary” function of human skin in relation to organ function still needs further investigation but important is that also changes in cutaneous mitochondrial oxygen consumption correlate with ODR changes in other organs and tissues [17]. The ODR from the dynamic measurement 6.3 mmHg s^−1^ in this example corresponds with previous data from healthy volunteers 5.8 ± 2.3 mmHg s^−1^ [29] .

In this paper we describe the case of clonidine given to a patient within a short period of time. This incidental finding occurred during an ongoing neurosurgery feasibility study. This patient group was chosen because in general we aim at hemodynamic stability during surgery over a longer time period. Therefore the incidental finding of an abrupt drop in cutaneous mitoPO_2_ after a bolus clonidine came clearly forward. In line with clinical observations the effects of clonidine administration resulted in initial vasoconstriction and subsequent vasodilatation after a couple of minutes. Using online monitoring we observed a direct decline in microvascular blood flow and velocity, followed by an increase in flow and velocity as measured by the O2C. With the change of flow and velocity a decrease in the oxygen supply to the tissue is expected. In consequence a decrease in mitoPO_2_ of 30 mmHg is observed as measured by the COMET.

However, interestingly, the capillary-venous oxygen saturation measured by the O2C did not show any decrease in the minutes following clonidine administration. Two main phenomena could explain the unchanged capillary-venous oxygen saturation. First, the SO_2_ is measured with the absorbance of visible light; the velocity and flow are measured with the hemoglobin laser doppler frequency shift. The different wavelengths of light, giving a different tissue penetration and therefore measurement compartment [30, 31], used in these techniques may explain why a difference in flow but not of SO_2_ after clonidine administration could be observed. A second explanation could be a total stop flow of some capillaries. The part of the capillary tree without flow does not contribute to venous-capillary saturation. Thus, the oxygen extraction in the measurement volume stays the same. For the COMET measurements heterogeneity of the oxygen content in the measurement volume could be demonstrated [8]. Therefore a heterogeneous bimodal distribution could explain the decrease in flow and a constant oxygen capillary venous saturation. The reader should keep in mind that for practical reasons the O2C and COMET measurements were performed in close proximity of each other, but not in exactly the same area of the skin. However, we do think that for a valid comparison of the measurements the fact that SO_2_ and mitoPO_2_ were measured at the same depth in skin is of more importance. Based on our findings it is clear that measuring oxygen availability directly at cellular level provides complementary data and new insight.

While COMET is the first clinical device for measuring mitochondrial oxygen and oxygen consumption, the used technology has some limitations. Currently the typical application time of the ALA on the skin is 4 h. This makes the measurement technique not yet applicable in acute situations. Furthermore, the combination of topical ALA administration and the green excitation light cause a very shallow measurement depth. While this does enable the oxygen consumption measurements, the oxygen measurements become more sensitive to tissue heterogeneity and background light.

Till now the feasibility of measuring mitoPO_2_ and mitoVO_2_ with PpIX-TSLT was tested in healthy volunteers [29] and is currently further evaluated with COMET in the perioperative setting. However, the original main development idea of COMET was the in vivo determination of aspects of mitochondrial (dys)function in critical illness. Indeed, in the laboratory setting the effectiveness of this technique in determining mitochondrial function under septic circumstances could be demonstrated [16].

Furthermore, mitochondrial oxygen measurements could potentially provide a new physiological transfusion trigger for decision-making in transfusion medicine. In a very recent animal study we have shown that mitoPO_2_ can be used as an early detector of reaching the individual limit of hemodilution before changes in systemic oxygen consumption and lactate levels occur [27]. If this concept can be translated into the anemic human situation, it is indeed potentially an individual physiological parameter to guide blood transfusions. The technique used in COMET is not limited to measurements in skin, since ALA can be administered systemically [32, 33]. Therefore, endoscopic or intraoperative measurement of mitoPO_2_ is technically feasible but such attempts should always take into account extensive safety considerations related to potential photodynamic toxicity.

## Conclusion

This report provides a description of the novel COMET measurement system. The enhanced protoporphyrin IX concentration in the skin is used as endogenous oxygen-sensitive probe. The method gives the possibility to measure cellular oxygen availability and the oxygen disappearance rate at the bedside on a mitochondrial level. In the future the COMET could play a role in clinical practice to assess tissue viability, to manage oxygen transport, and to recognize and possibly to treat mitochondrial inhibition in critically ill patients. Furthermore it potentially can be used as an individual blood transfusion trigger and may enable testing mitochondrial effects of pharmaceutical substances research.
